# Correction: Xin et al. Dexras1 Induces Dysdifferentiation of Oligodendrocytes and Myelin Injury by Inhibiting the cAMP-CREB Pathway after Subarachnoid Hemorrhage. *Cells* 2022, *11*, 2976

**DOI:** 10.3390/cells13231975

**Published:** 2024-11-29

**Authors:** Yuanjun Xin, Jie Chen, Hongxia Zhang, Robert P. Ostrowski, Yidan Liang, Jun Zhao, Xiang Xiang, Fuming Liang, Wenqiao Fu, Hao Huang, Xintong Wu, Jun Su, Jiewen Deng, Zhaohui He

**Affiliations:** 1 Department of Neurosurgery, The First Affiliated Hospital of Chongqing Medical University, 1 Friendship Road, Chongqing 400016, China; 2017110310@stu.cqmu.edu.cn (Y.X.); 13657672452@163.com (H.Z.); liangyidan0109@126.com (Y.L.); zhaoj_yy@163.com (J.Z.); daisuke0620@163.com (X.X.); 18180192008@163.com (F.L.); fwqmdical@163.com (W.F.); haosama666666@163.com (H.H.); wxt_0730@163.com (X.W.); sujun0724@163.com (J.S.); hcthsaya@gmail.com (J.D.); 2 Laboratory of Skeletal Development and Regeneration, Institute of Life Sciences, Chongqing Medical University, Chongqing 400016, China; jiechen@stu.cqmu.edu.cn; 3 Department of Experimental and Clinical Neuropathology, Mossakowski Medical Research Institute Polish Academy of Sciences, 02-106 Warsaw, Poland; rostro2104@yahoo.com

## Error in Figure

In the original publication [1], there was a mistake in Figure 8D. The authors accidentally inserted the image of SAH + LV-scramble in Figure 6D into the Figure 8D SAH + 8-Bromo-cAMP image when authors were drawing. The corrected version of Figure 8 appears below. 

The authors state that the scientific conclusions are unaffected. This correction was approved by the Academic Editor. The original publication has also been updated.

## Figures and Tables

**Figure 8:**
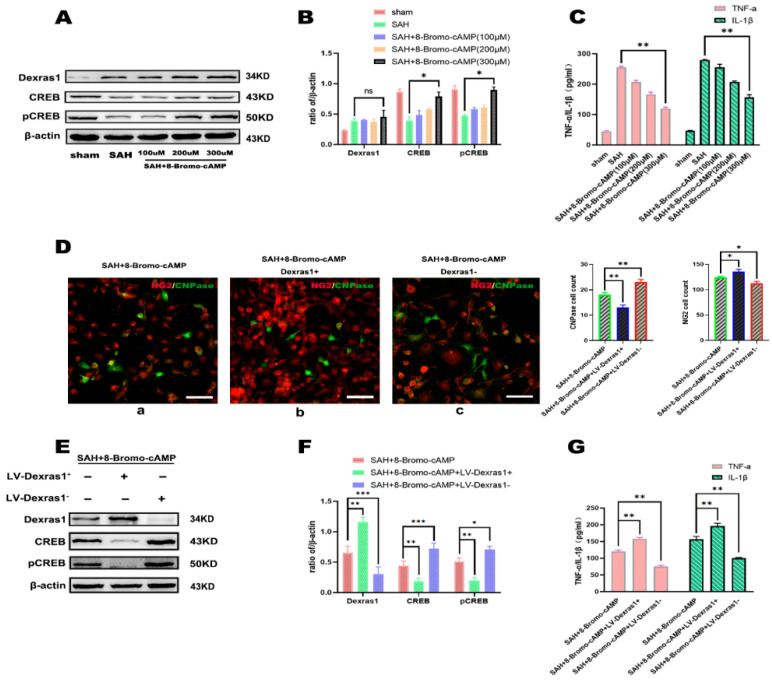
(**A**) Representative Western blots of Dexras1, CREB, and pCREB expression after in vitro subarachnoid hemorrhage with the activation by different 8-Bromo-cAMP sodium salts; (**B**) Western blot analysis of Dexras1, CREB, and pCREB. Results are percentages relative to β-actin levels. (* *p* < 0.05); (**C**) the results of ELISA used to detect IL-1β and TNF-α levels, (** *p* < 0.01); (**D**) immunofluorescence detection of oligodendrocyte precursor cell line differentiation after in vitro subarachnoid hemorrhage in each group, co-culture of SAH+8-Bromo-cAMP group neurons and oligodendrocyte cells treated with IGF−1 (a), co-culture of SAH+8BMP+Dexras1^+^group neurons and oligodendrocyte cells treated with IGF−1 (b), co-culture of SAH+8-Bromo-cAMP+Dexras1^−^group neurons and oligodendrocyte cells treated with IGF−1 (c), OPCs (NG2, red), OLGs (CNPase, green). Scale bars: ea = 25 μm. (* *p* < 0.05, ** *p* < 0.01); (**E**) representative Western blots of Dexras1, CREB, and pCREB after 8-Bromo-cAMP sodium salt treatment combined with Dexras1 overexpression or knockdown after subarachnoid hemorrhage in vitro; (**F**) Western blot semi-quantitative analysis of Dexras1, CREB, and pCRE. Results are percentages relative to β-actin levels. (* *p* < 0.05, ** *p* < 0.01, *** *p* < 0.001) (**G**). ELISA results show IL-1β and TNF-α levels. (** *p* < 0.01).
